# Neuroprotective Effect of Thymoquinone, the *Nigella Sativa* Bioactive Compound, in 6-Hydroxydopamine-Induced Hemi-Parkinsonian Rat Model

**Published:** 2014

**Authors:** Reza Sedaghat, Mehrdad Roghani, Mohsen Khalili

**Affiliations:** a *Department of Pathology and Anatomy, School of Medicine, Shahed University, Tehran, Iran.*; b *Neurophysiology Research Center, Shahed University, Tehran, Iran.*

**Keywords:** Thymoquinone, Parkinson’s disease, 6-Hydroxydopamine, Oxidative stress

## Abstract

Parkinson disease (PD) is the most common movement disorder with progressive degeneration of midbrain dopaminergic neurons for which current treatments afford symptomatic relief with no-prevention of disease progression. Due to the neuroprotective property of the *Nigella sativa* bioactive compound thymoquinone (TQ), this study was undertaken to evaluate whether TQ could improve behavioral and cellular abnormalities and markers of oxidative stress in an experimental model of early PD in rat. Unilateral intrastriatal 6-hydroxydopamine (6-OHDA)-lesioned rats were daily pretreated p.o. with TQ at doses of 5 and/or 10 mg/Kg three times at an interval of 24 h. After 1 week, apomorphine caused contralateral rotations, a reduction in the number of neurons on the left side of the substantia nigra pars compacta (SNC) was observed, malondialdehyde (MDA) and nitrite level in midbrain homogenate increased and activity of superoxide dismutase (SOD) reduced in the 6-OHDA lesion group. TQ pretreatment significantly improved turning behavior, prevented loss of SNC neurons, and lowered level of MDA. These results suggest that TQ could afford neuroprotection against 6-OHDA neurotoxicity that is partly due to the attenuation of lipid peroxidation and this may provide benefits, along with other therapies, in neurodegenerative disorders including PD.

## Introduction

Parkinson’s disease (PD) is the most common movement disorder in population (1) that is characterized by debilitating motor abnormalities including resting tremor, muscle rigidity, difficulty in initiation of voluntary movements, and postural instability (2). Primary neuropathological hallmark of PD is the progressive degeneration of the nigrostriatal dopaminergic neurons (1). The neurotoxin 6-hydroxydopamine (6-OHDA) has been used to initiate degeneration of dopaminergic neurons and experimental parkinsonism in rodents (3). Following 6-OHDA injection, some behavioral, biochemical, and pathological hallmarks of PD develop (4). The toxic effects of 6-OHDA are due to enhanced oxidative stress burden, inflammatory processes and apoptosis (5). Mitochondrial dysfunction and increased oxidative stress are also responsible for neuronal loss in patients with PD (6). Although great advances have been achieved in the development of novel and safe agents to treat PD, to date, no pharmacological agent has convincingly demonstrated the ability to slow the progression of PD (7). 

Thymoquinone (TQ) is the bioactive constituent of the volatile oil of black seed (*Nigella sativa)* (8) with anti-inflammatory, anti-oxidant (9) and anti-tumor activity (8). TQ has a protective role against ethanol-induced neuronal apoptosis in primary rat cortical neurons (10) and protects PC12 cells against cytotoxic agents via attenuation of oxidative stress (11). TQ could also ameliorate neurodegeneration in frontal cortex after chronic toluene exposure in rats (12). Potential of TQ to protect dopaminergic neurons in cell culture against MPP+ and rotenone cytotoxicity has already been reported (13). Considering these beneficial effects, the present study attempted to investigate the neuroprotective effect of TQ in 6-OHDA rat model of early hemi-parkinsonism and to determine the role of oxidative stress.

## Experimental

Adult male Wistar rats (190-250 g; n = 50) (Pasteur’s Institute, Tehran, Iran) were housed three to four per cage in a temperature-controlled colony room under light/dark cycle with food and water available ad libitum. Procedures involving animals and their care were conducted in conformity with NIH guidelines for the care and use of laboratory animals. The animals were held in the colony room for at least two weeks before being tested. Only rats not showing any biased rotational behavior (net rotations less than 30/hour) following intraperitoneal injection of apomorphine hydrochloride (2 mg/Kg) (Sigma Chemical, USA) were selected for the present study. The animals were randomly divided into five groups: sham-operated group, thymoquinone10-treated sham-operated groups (Sham + Thymoquinone10), lesion group (6-OHDA) and thymoquinone-treated lesion groups (6-OHDA + Thymoquinone5 and 6-OHDA + Thymoquinone10). Unilateral intrastriatal 6-OHDA (Sigma Chemical, USA) injection (left side) was performed through a 5 μL Hamilton syringe on anesthetized rats (ketamine 80 mg/Kg and xylazine 10 mg/Kg, *i.p*.) using stereotaxic apparatus (Stoelting, USA) at the coordinates: L –3 mm, AP  9.2 mm, V   4.5 mm from the center of the interaural line, according to the atlas of Paxinos and Watson (14). At the end of injection, the needle was left in place for an additional 5 min and then withdrawn at a rate of 1 mm/min. The lesion group received a single injection of 5 μL of 0.9% saline containing 2.5 μg/μL of 6-hydroxydopamine-HCL (6-OHDA, Sigma Chemical, USA) and 0.2% ascorbic acid (W/V) at a rate of 1 μL/min. The sham group received an identical volume of ascorbate-saline solution. The 6-OHDA + thymoquinone5 and 6-OHDA + thymoquinone10 groups received the neurotoxin in addition to TQ p.o. (using rodent gavage) dissolved in propylene glycol (Merck, Germany) at doses of 5 and/or 10 mg/Kg respectively. TQ (Sigma Chemical, USA; purity > 97%) was daily administered from two days before surgery with an interval of 24 h (3). The third injection of thymoquinone was 1 h before surgery. 

*Behavioral testing*

The animals were tested for rotational behavior by apomorphine hydrochloride (2 mg/Kg, *i.p*.) one week before surgery (baseline) and after 1 week. The rotations were measured according to a method as described previously (3). Briefly, the animals were allowed to habituate for 10 min and then 1 min after the injection, full rotations were counted in a cylindrical container (a diameter of 33 cm and a height of 35 cm) at 10-min intervals for 60 min in a dimly-lighted room. Net number of rotations was defined as the positive scores minus the negative scores.

*Determination of midbrain MDA concentration*

The rats were anesthetized with ketamine (150 mg/Kg), decapitated, brains were removed, anterior third block of left midbrain was blotted dry, weighed, then made into 5% tissue homogenate in ice-cold 0.9% saline solution, centrifuged at 4 ºC, obtained supernatant was aliquotted, then stored at −80 °C until assayed (3). The MDA concentration (thiobarbituric acid reactive substances, TBARS) in the supernatant was measured as described before (3). Briefly, trichloroacetic acid and TBARS reagent were added to supernatant, then mixed and incubated at boiling water for 80 min. After cooling on ice, samples were centrifuged at 1000×g for 10 min and the absorbance of the supernatant was read at 532 nm. TBARS results were expressed as MDA equivalents using tetraethoxypropane as standard. 

*Measurement of midbrain SOD activity*

The supernatant of midbrain homogenate was obtained as described earlier. SOD activity measurement was according to previous works (3). Briefly, supernatant was incubated with xanthine and xanthine oxidase in potassium phosphate buffer (pH 7.8, 37 ºC) for 40 min and NBT was added. Blue formazan was then monitored spectrophotometrically at 550 nm. The amount of protein that inhibited NBT reduction to 50% maximum was defined as 1 nitrite unit (NU) of SOD activity. 

*Assay of midbrain nitrite concentration*

Supernatant nitrite content was assayed by the Griess method according to previous studies (15). Because NO is a compound with a short half-life and is rapidly converted to the stable end products nitrate (NO3 -) and nitrite (NO2–), the principle of the assay is the conversion of nitrate into nitrite by cadmium and followed by color development with Griess reagent (containing sulfanilamide and N-naphthyl ethylenediamine) in acidic medium. The total nitrite was measured by Griess reaction. The absorbance was determined at 540 nm with a spectrophotometer.

*Protein Assay*

The protein content of the supernatant was measured with Bradford method using bovine serum albumin (Sigma Chemical, USA) as the standard (16).

*Histological study*

Half of the animals in each group were randomly used for histological assessment. At the end of behavioral experiments, the rats were deeply anesthetized with a high dose of ketamine (150 mg/Kg) and perfused through the ascending aorta with 50-100 mL of 0.9% saline followed by 100-200 mL of fixative solution containing 4% paraformaldehyde in 0.1 M phosphate buffer (PB, pH 7.4) followed by 100 mL of 0.1 M PB containing 10% sucrose. Following perfusion, the brains were removed from the skull, blocks of forebrain and brainstem were prepared, and after final steps of preparation (immersion in 30% sucrose solution for 2-3 days), sections were cut at a thickness of 40 μm on a freezing microtome (Leica, Germany) and collected in PB (0.1 M). Every second section was Nissl-stained with 0.1% cresyl violet (Sigma chemical, USA). 

*Neuronal counting*

For each animal, mesencephalic sections (Interaural 2.9-4.2 mm) were examined by a method as described previously (17). Briefly, Nissl-stained neurons of the SNC were counted manually (Light microscopy; X400) using a superimposed grid to facilitate the procedure. At least two sections representative of each of four Paxinos-Watson planes (4.2, 3.7, 3.2, 2.97; Interaural) were examined by scanning the entire extent on each side. Counting was done blind to the treatments received. 

*Statistical analysis*

All data were expressed as mean   S.E.M. For the drug-induced rotational behavior, non-parametric Kruskall-Wallis test was used. Inter-group differences for values of Nissl-stained neurons for the injected side and biochemical assays were found out using one-way ANOVA followed by Tukey’s post-hoc test. In all analyses, the null hypothesis was rejected at a level of 0.05. 

## Results

**Figure 1:**
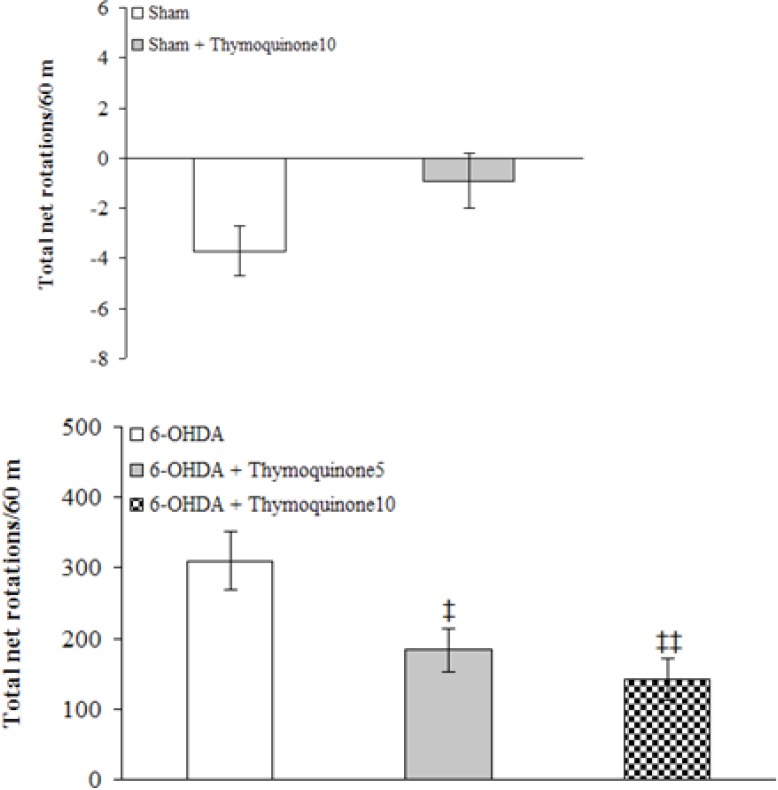
Total net number of rotations (mean ± S.E.M.) induced by apomorphine (2 mg/Kg, i.p.) after 1 week over a period of 60 min in sham (upper panel) and 6-OHDA-lesioned (lower panel) groups.

The beneficial effect of TQ was evaluated on apomorphine-induced rotations for a period of 1 hour (Figure 1). 

Note that the positive values indicate contralateral rotations. 6-OHDA stands for the neurotoxin 6-hydroxydopamine.

p < 0.05, ‡‡ p < 0.01 (versus 6-OHDA).

There were no-significant differences among the groups at baseline (before surgery). Statistical analysis of the total net number of rotations 1 week after the surgery showed that apomorphine caused a very significant contralateral turning in the rats of 6-OHDA group (p < 0.0001) and induced less significant rotations in 6-OHDA+ thymoquinone 5 and 6-OHDA + thymoquinone10 groups (p < 0.001 and p < 0.005, respectively) in comparison with Sham group. Moreover, both 6-OHDA + thymoquinone 5 and 6-OHDA + thymoquinone 10 groups showed a significant reduction of rotations (p < 0.05 and p < 0.01, respectively) when compared to 6-OHDA group.

**Figure 2:**
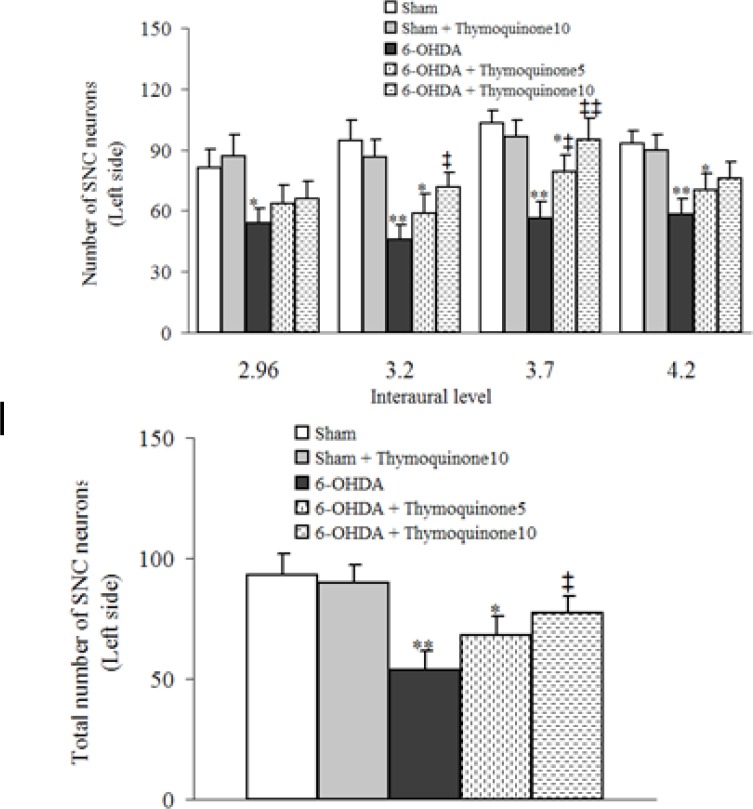
Total number of Nissl-stained neurons on the left side of substantia nigra pars compacta (SNC) at different interaural stereotaxic planes (upper panel) and its averaged number at all planes (lower panel) in different groups after 1 week post-surgery. 6-OHDA stands for the neurotoxin 6-hydroxydopamine. * p < 0.05, ** p < 0.01 (in comparison with Sham). ‡ p < 0.05, ‡‡ p < 0.01 (in comparison with 6-OHDA).

**Figure 3:**
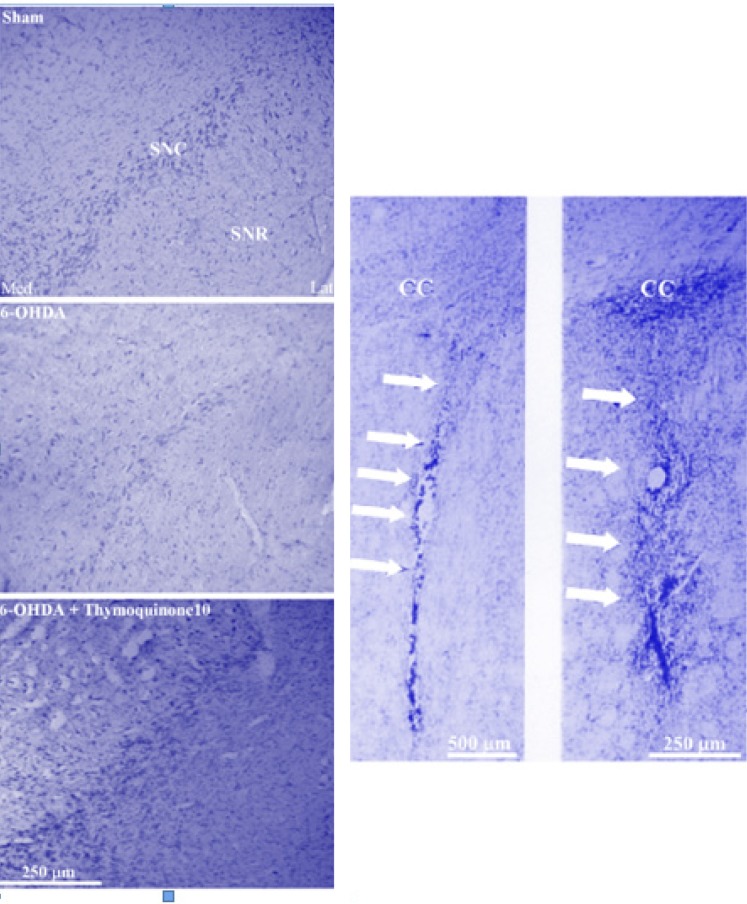
Photomicrograph of coronal sections through the midbrain showing Nissl-stained neurons in experimental groups (left panels) and injection site at low- and high-power magnifications in neostriatum (right panel). A severe reduction in the number of neurons in SNC was observed in the 6-OHDA lesioned group, but no such marked reduction was noted in the thymoquinone 10-treated lesioned group in comparison with Sham group. Scale bar = 250 μm (SNC and SNR = Substantia nigra pars compacta and pars reticulata respectively; CC=corpus callosum).

The results of histochemical studies (Figures 2 and 3) showed that there is no-significant difference between sham and sham+ thymoquinone10 regarding number of Nissl-stained neurons on the left side of SNC. Meanwhile, a significant reduction was observed in 6-OHDA group (p < 0.01) and a less significant reduction was noted for 6-OHDA + thymoquinone 5 (p < 0.05) and no such reduction was obtained for 6-OHDA + thymoquinone 10 group when compared to sham group. In addition, number of Nissl-stained neurons on the left side of SNS was significantly higher in 6-OHDA+ thymoquinone 10 versus 6-OHDA group (p < 0.05).

**Figure 4:**
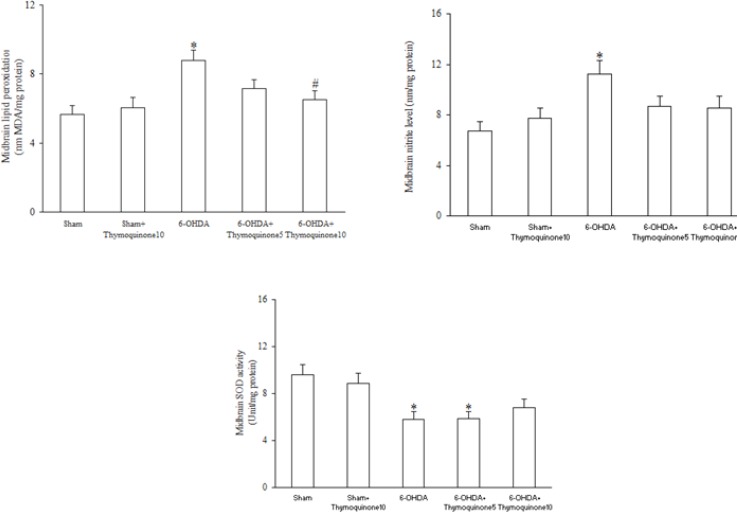
MDA content (top panel), nitrite content (middle panel) and superoxide dismutase (SOD) activity (bottom panel) in midbrain homogenate 6-OHDA stands for 6-hydroxydopamine. *p < 0.05 (versus Sham). #p < 0.05 (versus 6-OHDA).

Regarding midbrain lipid peroxidation and oxidative stress markers (Figure 4), 6-OHDA injection resulted in significant elevation of MDA (as a marker of lipid peroxidation) (p < 0.05) and nitrite content (p < 0.05) and a significant reduction of SOD activity (p < 0.05) and treatment of 6-OHDA-lesioned rats with TQ at a dose of 10 mg/Kg significantly attenuated only the increased MDA content (p < 0.05) with no-improvement of nitrite content and SOD activity.

## Discussion

In this study, we demonstrated that TQ at a dose of 10 mg/Kg significantly decreases apomorphine-induced rotations, attenuates loss of SNC neurons and lowers midbrain level of MDA in 6-OHDA-lesioned rats.

The selective loss of dopaminergic neurons in the SNC appears to be the direct cause of neurodegeneration in patients with PD (18). Also, 6-OHDA, which is commonly used for the induction of PD in animals, is believed to cause degeneration of dopaminergic neurons (4). The unilateral damage of the nigrostriatal dopaminergic system through intrastriatal injection of 6-OHDA is followed by a reduction in the striatal dopamine level and an upregulation of dopaminergic postsynaptic receptors at the same side. These changes produce a prominent functional and motor asymmetry that can be evaluated by dopaminergic agonists like apomorphine (19). These rotations are considered as reliable indicators of nigrostriatal dopamine depletion (20). In this study, a significant attenuation of the apomorphine-induced rotational behavior was observed in TQ-pretreated 6-OHDA-lesioned group after 1 week. The observed attenuation of rotational behavior in TQ-pretreated lesioned group could be attributed to possible protective effect of TQ against SNC neurodegeneration and maintenance of striatal dopamine at a level that is not accompanied with a marked rotational behavior. In other words, nigrostriatal neurons within SNC were mainly preserved in the presence of TQ against neurodegenerative effects induced by the neurotoxin 6-OHDA.

Free radicals are strongly involved in the toxicity of 6-OHDA-induced nigrostriatal lesions (21). Oxidative stress is important factor that could affect the survival of dopaminergic neurons in PD. Neurons mostly depend on energy produced by mitochondria and are simultaneously faced with high levels of reactive oxygen species (ROS) as well as increased levels of free iron, which can promote OH generation (22). Overload of the free radical formation may lead to cell death. Free radical scavengers may be helpful in prolonging survival time of dopaminergic neurons (23). In this respect, TQ could attenuate neuronal damage and loss through counteracting oxidative stress, possibly via regulating antioxidant defense system as well as inhibition of free radical generation (9, 24).

Inflammation in the brain is another causative factor in the pathogenesis of PD (25). Pro-inflammatory cytokines released from glial cells could stimulate nitric oxide production and exert a deleterious effect on dopaminergic neurons by activating receptors that contain intra-cytoplasmic death domains involved in apoptotic pathway (26). It has been shown that TQ has anti-inflammatory activity (27) and inhibits lipopolysaccharide-induced inflammatory processes (28). It is possible that TQ may have decreased the level of inflammatory mediators within the brain, which itself contributes to neuroprotection in 6-OHDA induced PD in rats, as observed in our study. Apoptosis is another factor that plays a critical role when cells are exposed to neurotoxins including 6-OHDA (29). TQ could suppress 6-OHDA-induced ROS generation and apoptosis through inhibition of the apoptotic cascade by increasing Bcl-2 expression (10). The results of another study has also suggested that TQ protects cultured dopaminergic TH immunoreactive cells from degeneration induced by MPP+ and rotenone (13) and this may have happened in our study.

In this study, midbrain SOD activity was lower in 6-OHDA group versus sham and TQ pretreatment at a dose of 10 mg/Kg was not able to significantly restore it. Since level of enzymes with antioxidant activity in brain regions like midbrain is lower than other tissues of the body, it is less likely that such treatments could improve it (30). In addition, it has been reported that TQ is not able to affect mitochondrial SOD level (31) and this may have occurred in our study. This fact itself requires further investigation.

Overall, the results of our study clearly suggest that TQ could afford neuroprotection against 6-OHDA neurotoxicity that is partially due to the attenuation of lipid peroxidation and this may provide benefits, along with other therapies, in neurodegenerative disorders including PD. However, further studies are required to understand its basic mechanisms of action.
